# Healthcare Experiences and Needs of Older Adults Living With HIV: A Qualitative Study

**DOI:** 10.1111/jocn.17538

**Published:** 2024-11-11

**Authors:** Marie Lund, Annelie J. Sundler, Ewa Carlsson Lalloo

**Affiliations:** ^1^ Faculty of Caring Science, Work Life and Social Welfare University of Borås Borås Sweden

**Keywords:** experiences, healthcare, human immunodeficiency virus, older adults, qualitative

## Abstract

**Aim:**

To explore the healthcare experiences and needs of older adults living with HIV in Sweden.

**Design:**

A phenomenological study using qualitative thematic analysis.

**Methods:**

Data were gathered through semistructured qualitative interviews and analysed using thematic analysis. A purposive sample of individuals ageing and living with HIV was recruited for this study.

**Results:**

A total of 22 participants aged 65 years and older, with a mean age of 75 years, participated in the study. Living with HIV meant a need to rely on consistent healthcare and treatment over the course of one's lifetime. The experiences of older adults were described under four themes: relying on access to health care, desiring involvement and shared decision‐making, the importance of trust and confidentiality and experiences of stigma in healthcare visits.

**Conclusion:**

Living and ageing with HIV involved recurring exposure and vulnerability in healthcare encounters, and experiences of being exposed to HIV‐related stigma, especially outside HIV clinics. Continuity and being seen as a person in healthcare encounters were important. Enhancing HIV knowledge alongside fostering supportive attitudes and approaches of healthcare professionals is critical for ensuring high‐quality and nondiscriminating health care.

**Implications for the Profession and/or Patient Care:**

Meeting the needs of older adults living with HIV requires a person‐centred approach, including active involvement and shared decision‐making between patients and healthcare providers.

**Impact:**

This study describes the healthcare experiences and needs of older adults, aged 65 years and older, living with HIV, a population that is not typically investigated in qualitative research. Living and ageing with HIV means a vulnerability where the attitudes of professionals and the active involvement of the patient become significant for quality care. The findings can help with the implementation of policies and practices for the care of older adults living with HIV.

**Reporting Method:**

The Consolidated Criteria for Reporting Qualitative Studies (COREQ) was used.

**Patient and Public Contribution:**

Patient representatives were involved in discussions on data collection and the development of the interview guide.

**Summary**

- What does this paper contribute to the wider global clinical community?
  ○ This study emphasises how people living with HIV need to rely on consistent healthcare and treatment over the course of their lifetime, indicating recurring exposure and vulnerability in healthcare encounters.
  ○ Active involvement and shared decision‐making with individuals in HIV care, together with professionals' attitudes and approaches, are significant for high‐quality and nondiscriminating person‐centred healthcare.

## 1 Introduction

This study contributes to the understanding of the healthcare experiences and needs of older adults, aged 65 years or older, living with human immunodeficiency virus (HIV), as the population in this study is not typically investigated, especially in qualitative research. This knowledge is crucial for guiding person‐centred healthcare initiatives aimed at supporting capacity and promoting healthy ageing among older adults living with HIV.

## 2 Background

Owing to advancements and increased accessibility of antiretroviral treatment (ART), the life expectancy of people living with HIV has increased significantly over the last 25 years (Trickey et al. 2023). This has resulted in a growing number of older adults living with HIV. As effective ART eliminates the risk of sexual transmission (Cambiano et al. 2019) and reduces symptoms, HIV is currently considered a chronic infection (Gisslén et al. 2017). In response to improved survival rates and better global access to antiretroviral therapy (ART), UNAIDS has raised its global testing, treatment and viral suppression targets from 90% to 95%. These include ensuring 95% of people living with HIV know their HIV status, 95% of people who know their status are receiving treatment and 95% of people on HIV treatment have a suppressed viral load. It also includes a target of zero discrimination (UNAIDS 2015a). HIV continues to exert a long‐term impact on individuals' health and healthcare needs as multimorbidity becomes increasingly prevalent among older adults living with HIV (Guaraldi et al. 2018; DerSarkissian et al. 2020). They have a higher prevalence of dyslipidaemia, hypertension, psychiatric disorders and diabetes than the general population (Belaunzaran‐Zamudio et al. 2020). There is also a higher risk of various cancers compared to the general population, such as Kaposi sarcoma, non‐Hodgkin lymphoma and cancers of the cervix and anus. This increased cancer risk is primarily due to HIV‐induced immunosuppression, coinfections with oncogenic viruses, and a high prevalence of behavioural risk factors such as smoking (Mahale et al. 2018). Additionally, the necessity for ongoing medical treatment can be a source of concern, as it involves the responsibility of adhering to daily medication regimens and harbouring fears about future health uncertainties, such as not being able to take care of oneself (Davis et al. 2023). Thus, the demand for a range of healthcare services has grown, presenting challenges in ensuring the delivery of high‐quality clinical care (Kokorelias et al. 2023).

HIV‐related stigma entails encountering negative attitudes or moral judgements and rejection from others when a person break perceived socially accepted norms (Goffman 1990). This, in turn, has detrimental effects on health and exacerbates health disparities. Adults living with HIV report that even if they had positive experiences from healthcare encounters, most individuals have faced stigma in such interactions, for instance related to healthcare professionals fear for transmission (Brinsdon, Abel, and Desrosiers 2017). Participants in that study reported that a strategy to manage this stigma was to be selective in their disclosure of ones HIV status. Additionally, older adults living with HIV face a heightened risk of encountering multiple stigmatisations, including both HIV‐related stigma and ageism. Ageism is a significant concern as it intersects with HIV‐related stigma, leading to compounded health risks and barriers to care (Hsieh et al. 2022). Studies also show that HIV stigma by healthcare providers is associated with low awareness about transmission routes and effective HIV treatment (Geter, Herron, and Sutton 2018; Tavakoli et al. 2020). These anticipated attitudes from healthcare providers may pose a barrier to HIV prevention and access to care and treatment (Relf et al. 2021). This can also lead to decreased adherence to ART and a reluctance to seek medical care (Pantelic et al. 2019). This stigma creates structural barriers to HIV prevention and treatment efforts, particularly for marginalised groups.

The definition of older adult is typically based on chronological age and varies across contexts. For adults living with HIV, 50 years is a commonly used threshold due to evidence of poorer immune recovery, higher risk of comorbidities and reduced survival rates (Sánchez‐Conde et al. 2019). With improved global access to ART and increased survival rates among individuals living with HIV, the focus has shifted towards understanding how HIV affects an individual's quality of life (Lazarus et al. 2016). To address the needs of older adults living with HIV and promote healthy ageing, it is imperative to gain a comprehensive understanding of their healthcare experiences. Such an understanding is essential for supporting lifelong health and well‐being. However, the current research on older people, aged 65 years or older and living with HIV are limited and there remains a gap in knowledge regarding the healthcare experiences and needs of this population. Specifically, there is a notable lack of qualitative studies. Therefore, conducting research describing the experiences of living and ageing with HIV among individuals aged 65 years and older is essential to fill this knowledge gap and inform targeted interventions to improve healthcare outcomes for this population. To avoid categorising middle‐aged individuals as elderly, this study sets the age threshold for older adults aged 65 and above, a threshold commonly used internationally and in Sweden and for defining older adults.

## 3 The Study

### 3.1 Aim

To explore the healthcare experiences and needs of older adults living with HIV in Sweden.

## 4 Methods

### 4.1 Design

This was a phenomenological study using qualitative thematic analysis. The guideline for Consolidated Criteria for Reporting Qualitative Studies (COREQ) was used (Tong, Sainsbury, and Craig 2007), see File S1. A phenomenological approach was employed to explore older adults' lived experiences, providing a deep understanding of how they perceive and make meaning of the phenomenon in focus. To analyse these experiences, a qualitative thematic analysis rooted in phenomenology (Sundler et al. 2019) was conducted, allowing for a detailed exploration and description of the meanings attributed to the studied phenomena.

The phenomenological descriptive approach used is grounded in a human science perspective, with an interest in the lifeworld and human intersubjectivity, including the interconnectedness of mind, body and spirit (Sundler et al. 2019; Dahlberg, Dahlberg, and Nyström 2008). The descriptive tradition of phenomenology originates from the writings of Husserl and was further developed by Merleau‐Ponty. Phenomenology involves understanding our subjectiveness, including who we are, our resources and skills, as they manifest in our lifeworld, social situations and relationships. This approach can be used to understand lifeworld phenomenon such as healthcare experiences. Consequently, it has implications for both data collection and analysis, requiring openness to the phenomenon and awareness of the researcher's preunderstanding of the studied phenomenon (Dahlberg, Dahlberg, and Nyström 2008). Therefore, researchers must have critical and reflective attitudes throughout the research process.

### 4.2 Study Setting and Recruitment

Older adults living with HIV in Sweden were recruited from HIV clinics and organisations between September and December 2022.

In Sweden, approximately 8500 people live with HIV of the total population of 10.5 million inhabitants. Of people with HIV 22% are aged 60 years or older. Most individuals living with HIV (98%) are receiving ART, have undetectable viral load (95%) and are followed up by a specialised HIV clinic funded through taxation (Carlander and Mattsson 2022). According to a Swedish legislation (Sveriges Riksdag [The Swedish Parliament] 2004:168), all associated costs are covered by the state. The legislation classifies HIV as a public health hazard among several other infections, and individuals living with HIV must avail themselves of the care and treatment provided at HIV clinics, exercise caution and take measures to prevent transmission to others. Additionally, they are required to disclose their HIV status in situations where there is a risk of HIV transmission (Sveriges Riksdag [The Swedish Parliament] 2004:168). The rules of conduct outlined in the legislation are tailored by the treating physician to suit the individual, considering factors such as HIV treatment outcomes and the obligation to disclose can thus be removed for individuals with well‐treated HIV (Folkhälsomyndigheten [Public Health Agency of Sweden] 2019).

Information regarding the study was distributed on the official website of University of Borås, to ensure a divers and representative sample of participants from different regions in Sweden. All HIV clinics for adults and HIV organisations in Sweden were contacted by the researchers and informed of the study. Information about the study was then spread digitally on webpages and social media, with contact information for the researchers.

Healthcare professionals at the HIV clinics identified eligible participants and provided them with information about the study and invitations to participate. The number of individuals asked for interest was not documented. If a person was interested in participating and consented to be contacted, one of the researchers contacted them via telephone. Three participants contacted the researchers themselves. When contacting possible participants, the researchers again gave them information about the study and allowed them to ask questions. Among 29 interested individuals, three did not respond when the researchers tried to contact them and four declined further participation after receiving more information about the study. In total, 22 participated in the interviews.

### 4.3 Inclusion and Exclusion Criteria

A purposive sampling strategy was used to include people ageing and living with HIV. The inclusion criteria were adults living with HIV in Sweden, aged 65 years or older. The exclusion criteria were older adults living with HIV for < 6 months or individuals diagnosed with dementia, as the researchers were unable to adequately address the ethical and practical challenges necessary to include individuals newly diagnosed with HIV or with cognitive impairments in a manner that respects their rights and dignity while ensuring the study's validity.

For the recruitment of older adults with varying healthcare experiences, all eligible patients were invited to participate. To minimise selection bias, recruitment focused on individuals willing to be interviewed and share their lived experiences. In addition, there was ongoing dialogue between researchers, clinicians and HIV organisations to ensure a diverse and representative sample. Towards the end of the recruitment period, we noted a shortage of individuals with complex healthcare needs. Despite additional efforts to recruit more participants with home care service for example, none of these individuals provided informed consent for participation.

### 4.4 Data Collection

Semistructured interviews were conducted to collect data. A semistructured interview guide was developed with open‐ended questions about individual experiences of health care and needs, in addition to follow‐up questions such as ‘Can you tell me more?’ and ‘What do you mean by that?’ The interview guide was developed in cooperation with the Public Health Agency of Sweden officials and with reference persons from civil society and HIV organisations. The research team evaluated the interview questions after the first two interviews and determined them to be effective, so no modifications were made. Demographic information was voluntary to answer, and was not recorded, as suggested by the reference persons.

All the researchers conducted interviews between October 2022 and January 2023. All researchers were trained in qualitative interviews. ML and ECL conducted most of the interviews. All interviews were conducted in Swedish at a time and location convenient for the participants. The interviews were conducted by telephone (*n* = 19), face‐to‐face or audio‐recorded and transcribed verbatim by the researchers. The participants could choose to be interviewed either at a mutually agreed‐upon location or via telephone. Although no reasons for choosing the telephone option was asked for, several participants mentioned that it was more convenient for them. The demographic information was documented by the researcher. Each interview lasted from 25 min to 1 h and 27 min, with an average length of 48.5 min. The quotes from the interviews used to illustrate the findings were translated into English.

### 4.5 Data Analysis

Data were analysed thematically using inductive and data‐driven analyses focusing on the meanings in the data. This method involves a movement between the whole text and parts of the text, where themes are developed based on patterns of meanings that emerge (Sundler et al. 2019). The researchers used their expertise and knowledge to understand the individual experiences and needs in health care. ML, a female registered nurse, holds a master's degree in healthcare sciences. AS, also a female registered nurse, is a professor in healthcare sciences with extensive qualitative research experience. ECL, a female registered nurse with a PhD in healthcare sciences, with experience in HIV research. All three are employed at the same academic institution.

Initially, all researchers read the text from the transcribed interviews repeatedly and with an open mind to familiarise themselves with the content of the data. Then the first author focused the reading to understand the meanings of the experiences with an awareness of the researchers preunderstanding. The meanings identified from the interviews were described in short notes and further summarised in a short text. By comparing similarities and differences in meanings, patterns emerged, and the text was further developed by ML and ECL and organised into preliminary themes. As the iterative process continued, the analysis and themes were reviewed and discussed by all researchers and their preunderstanding about the phenomenon was reflected upon. Researcher must have an openness towards data to let unexpected meanings to emerge. Openness also includes an awareness of researchers' assumptions and beliefs to minimise its influence on the analysis and the emerging themes. During the analysis process, the researchers went back and forth between original interview data and researchers' description of themes and meanings. During the process, themes were reflected upon and refined by all researchers. The analysis resulted in four themes. To strengthen the trustworthiness of the analysis and illustrate the meanings expressed by the participants, the final themes and related descriptive texts were illustrated with quotes. Quotations were translated from Swedish by the researchers and reviewed by a professional language editing service.

### 4.6 Ethical Considerations

This study was approved by the Swedish Ethical Review Authority (2022‐02203‐01) and followed the ethics for medical research on humans according to the Declaration of Helsinki (World Medical Association [WMA] 2018). All the participants received oral and written information regarding the study. The participants also received information about voluntary participation and the possibility of withdrawing their participation whenever they wanted, without any further questions. They were also guaranteed that nonparticipation would not impact their present or future health care. All participants provided oral or written informed consent to participate, as documented by the researchers.

### 4.7 Rigour and Reflexivity

The rigour and validity of the current study are linked to the methodological principles of openness, a reflective approach and consciously questioning preunderstanding, which is further discussed in terms of reflexivity, credibility and transferability (Sundler et al. 2019).

In striving for reflexivity, a reflective approach was used throughout the research process, involving constantly questioning and discussing the researchers' pre‐understanding. To ensure credibility, the research process must be transparent and easy for readers to comprehend. Therefore, the researchers have attempted to describe the steps involved in the analysis and development of themes clearly. The themes were repeatedly compared with the original text in the interviews to ensure that all meanings from the data were included in the analysis and the themes were described. Moreover, the analysis was reviewed and discussed among the researchers to ensure that the identified themes accurately captured the meanings derived from the data and that these meanings were described in a logical and clear manner, illustrated with quotes to validate the content and interpretations. Transferability is related to the relevance and usefulness of the findings and is thus linked to the results of the research process.

## 5 Findings

### 5.1 Characteristics of Participants

The 22 participants included in the study were aged 65 years or older, with a mean age of 75 years and 14 identified themselves as men and 8 as women. Not all participants answered every question about demographics. They had varied experiences of how long they had lived with HIV, ranging from 2 to 30 years. All the participants were on ART. The participants originated from Sweden (*n* = 16) and abroad (*n* = 6), such as other European or sub‐Saharan countries. They currently lived in both rural and urban areas of Sweden, from south to north. None of the participants reported living in residential care or expressed a need for home care. Overall, the participants' characteristics varied in terms of age, sex, sexual identity, family status, way of HIV transmission, years of living with HIV, life and work experience, socioeconomic status, living conditions, native language and experiences of migration.

### 5.2 Healthcare Experiences and Needs in Old Age When Living With HIV

**FIGURE 1:**
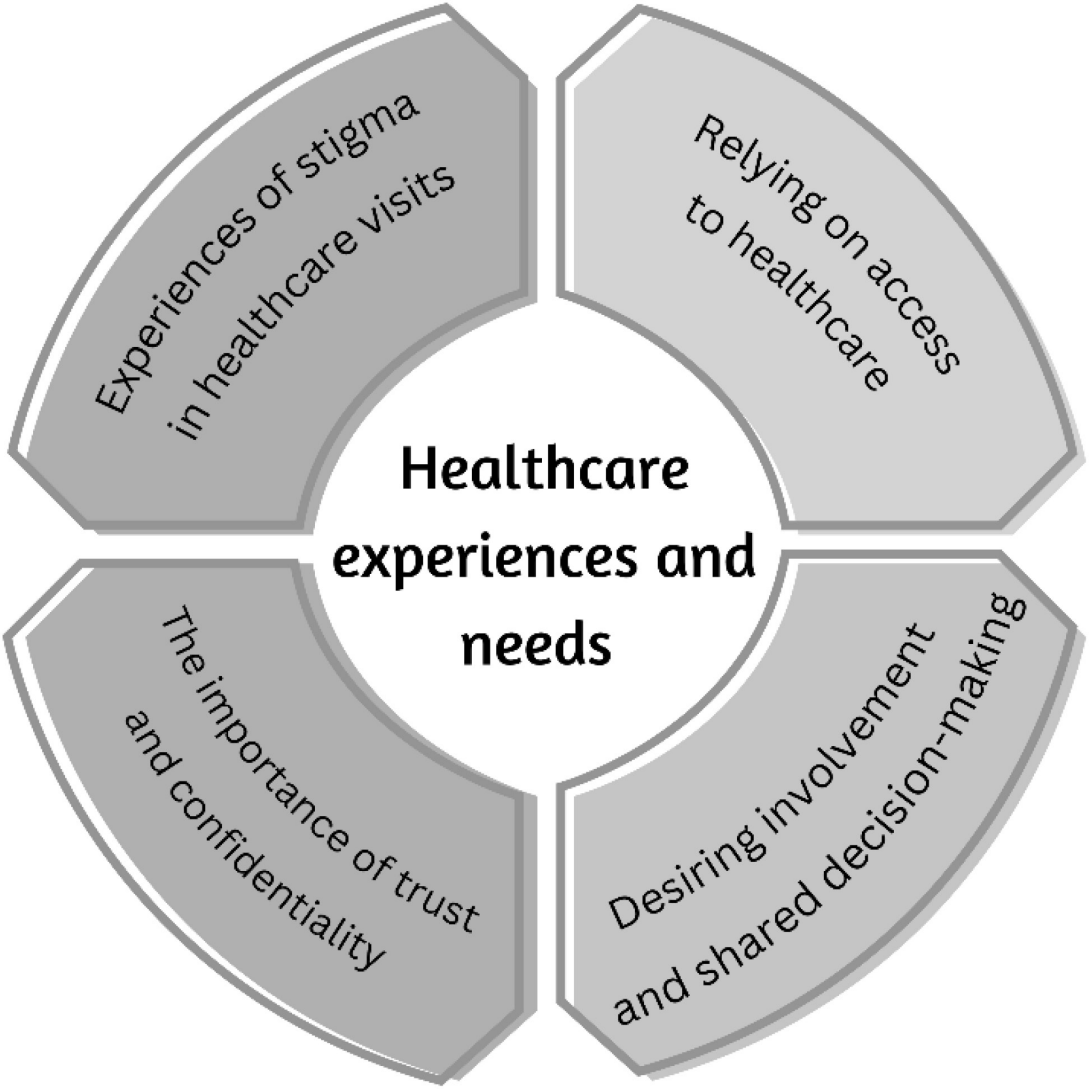
Overview of the four themes describing the healthcare experiences and needs among older adults living with HIV.

Living with HIV meant living with consistent healthcare and treatment over the course of one's lifetime. This was connected to managing a chronic infection and an intrinsic desire for optimal health alongside legal obligations related to HIV. Against this background, older adults' healthcare experiences and needs when living with HIV are described under four themes (Figure 1):

- Relying on access to healthcare
- Desiring involvement and shared decision‐making
- The importance of trust and confidentiality
- Experiences of stigma in healthcare visits

#### 5.2.1 Relying on Access to Health Care

Relying on access to healthcare and treatment was an important aspect for older adults living with HIV, as living with HIV involved consistent health care, with regular visits to specialised HIV clinics for checkups and ongoing care. In addition, living with HIV influenced interactions with other healthcare services and the need for accessible health care. This need was expressed deeply, irrespective of whether it was related to HIV or other symptoms and illnesses.

Overall, healthcare services were experienced as well‐functioning, expressed as ‘I often hear myself praising Swedish healthcare’ (3). HIV clinics were most experienced as being specifically well‐functioning.

What if I had some power and I could clone things? Then, I would clone the healthcare system together with the HIV clinic so it would function like them. (16)

However, experiences about encountering barriers to accessing healthcare services were described, especially outside HIV clinics. Not having access to necessary healthcare was inconvenient and perceived as a barrier to health and well‐being. Not receiving adequate care and treatment or facing long waiting periods when trying to schedule visits resulted in a feeling of the need to struggle. This was illustrated by one of the older adults: ‘You really have to be very active yourself’ (3). This made them feel frustrated and they had a sense of having to personally manage and take charge of their own healthcare needs.

The practice of referring older adults to primary care services instead of having direct access to a healthcare contact at an HIV clinic was a subject of concern. However, navigating multiple healthcare facilities was challenging for most of the older adults. From their perspective, the healthcare system was perceived as fragmented, with deficiencies in coordination and continuity. They narrated how they needed to be the link for information between different units. This contributed to a sense of not feeling supported and being able to rely on information transfer and the coordination of healthcare services.

I shouldn't have to call one hospital to tell them what is said at another. (17)

Other inconveniences arose when dealing with multiple healthcare facilities. The need for the repetition of information about their health status and information from their medical records and having different healthcare contacts for different health concerns fostered frustration and a sense of being shuffled between facilities without a cohesive approach to care.

#### 5.2.2 Desiring Involvement and Shared Decision‐Making

The older adults strongly desired to be acknowledged as individuals fully capable of actively participating in and overseeing their own care and treatment, both for HIV and other symptoms. This was enabled when healthcare professionals addressed their immediate needs during healthcare contacts.

If I have a problem or if I am sick and I am failing, it is important that I am welcome there. (7)

The participants expressed gratitude for having access to free ART, but specifically for the healthcare professionals' expertise at HIV clinics in managing HIV. This appreciation was illustrated, for instance, in collaboratively tracking and discussing treatment outcomes or jointly reviewing laboratory results with healthcare professionals.

So, I can see my HIV outcomes, they follow the curve exactly right there. I follow the curve each time, you know. There are two outcomes: What's it called? Resistance to disease, right? And then I have the other outcome, which is the viral load. On this curve (viral load), it's marked as the lowest. So, the treatment is going really well; no problems. (19)

The feeling of control and involvement in their health, care and treatment processes was advocated for. Participation in care processes was described as essential and fostered confidence in the nonrisk of transmitting HIV when having no viral loads. When actively involved and convinced by the test results, immense relief was described, which also relieved the obligation to disclose one's HIV status to others, such as healthcare providers.

At the HIV clinics, healthcare professionals were commonly perceived as ‘interested in their lives’ (18). There was an expressed need to be treated like this, regardless of the healthcare facility.

To see me and not the HIV‐person, but a person living with HIV, a patient who needs help, someone you need to talk to, someone you need to listen to. (22)

When professionals demonstrated an understanding of individuals' unique circumstances and offered personalised care, they fostered confidence in the healthcare system.

Older adults living with HIV frequently found themselves uncertain about the origins of eventual symptoms, whether they were related to HIV or ageing. This uncertainty made them rely on the expertise of healthcare providers as they sought clarification and guidance.

I'm very scared first and foremost because they don't seem to know; they don't know which illnesses are a result of HIV. They're guessing, which makes me a little… if they don't know, how am I supposed to know? Because what happens most of the time is that I'm the one telling them, instead of the other way around… and they're like, ‘I don't know anything about HIV, I don't know if this is because of HIV.’ So, that part actually scares me; I'm a bit afraid of that. Aging. (22)

Consequently, some were concerned about their advancing age and potential health challenges in the future. For instance, some expressed worries about deteriorating health, the risk of geriatric symptoms and cognitive impairment, which could lead to an inability to manage medications independently. They harboured fears of losing control, needing residential or home care services and abandoning their independence, thereby losing autonomy.

It might not work so well in elderly care and home care and I need to take my pill every day, which is very important, and they might not be so exact with it. And then, it could lead to what the doctor says, that if you don't take the pill, it (the viral load) could come back. (13)

Instead of worrying about their advancing age and potential healthcare challenges, some expressed a sense of solace by saying, ‘I don't think about that at all’ (15).

#### 5.2.3 The Importance of Trust and Confidentiality

Living with HIV meant a particular vulnerability in healthcare contacts. It was important to maintain confidentiality within the healthcare system and trust healthcare professionals. HIV was described by some older adults as something impossible to put behind in healthcare contacts. Some disclosed that they did not have any problems with being open about their HIV status.

Yes, I had to tell them that I had it, but that was it, she just said, “Okay, then I'll write this down, I'll note that.” (1)

Others talked about their profound commitment to safeguarding others from potential risks of transmission. Consequently, they commonly disclosed their HIV status voluntarily even when not required to do so. The choice of when to disclose their HIV status was considered to lie with themselves. However, they encountered ignorance from healthcare professionals, who believed that they were obligated to disclose their HIV status in all situations, regardless of whether the HIV was well‐adjusted:

But the doctor insisted that “You should inform about your HIV status every time you seek medical care.” But it's a bit strange; should I shout that I am HIV positive every time I enter a healthcare facility? (16)

Additionally, they expressed fear of disclosing their HIV status because of the risk of healthcare professionals not maintaining confidentiality. In some situations, this fear hindered individuals from seeking necessary health care, particularly when living in rural areas where they were not anonymous to the healthcare professionals:

Because I think that I might meet someone I know when I'm sitting there. … I would never just go to the lab and take blood tests. Then that person (an acquaintance) sees exactly what tests I am supposed to take. (9)

Additionally, the situation was complicated by their personal relationships with these professionals, which intensified their desire to keep their HIV status private.

Familiarity with healthcare professionals was valued in other situations. When individuals felt comfortable with others, this sense of acquaintance could lead to greater comfort and ease during visits.

I usually say that at the HIV clinic, they are like my friends, and we have very good contact. It means a lot, of course…. I feel at home there. For me, keeping the same doctor is important. (2)

Specifically, HIV clinics were highly valued for consistent encounters with the same healthcare provider, thus contributing to the creation of a welcoming and caring atmosphere. This created trust in knowing that one would be cared for with empathy, understanding and the needed support would be received.

Some expressed concerns about the future needs for elderly care and did not know how healthcare providers might react upon learning about their HIV status, potentially resulting in suboptimal care. Older adults strongly advocated that they wanted to maintain their independence for as long as possible. They preferred to rely on family support rather than home care services.

#### 5.2.4 Experiences of Stigma in Healthcare Visits

Older adults living with HIV experienced varying degrees of stigma, sometimes leading to discrimination during healthcare visits. Past negative experiences of stigma influenced their future healthcare contacts and anticipation of discriminatory attitudes.

Individuals who have lived with HIV for years could provide a timeline of attitudes towards people living with HIV. Some had observed a significant shift over time, transitioning from initial encounters marked by fear and caution, such as instances where ‘they wouldn't touch me without gloves and protective wear’ (19), to a gradual increase in awareness and understanding surrounding HIV. Additionally, individuals with experience abroad noted that healthcare systems in other countries often exhibited even higher levels of stigma towards HIV.

Previous encounters shaped expectations of encountering stigma. Recent healthcare encounters involving judgements or preconceptions regarding HIV were common among older adults. Some experienced being approached with excessive caution, indicating fear among healthcare professionals.

He was going to pull a tooth, but suddenly he put on many plastic gloves and stuff, and a face mask… No, and I didn't care about it either. I let him do his thing; there's no point in commenting. Yes, I do get a bit angry. Yes, I get slightly upset. Frankly. (18)

This over‐precaution made older adults feel stigmatised, thereby increasing their anxiety and discomfort in healthcare.

At times, individuals faced more subtle signs of stigmatisation in attitudes, which affected their healthcare experiences. These smaller signs, which are not always overt, could manifest through discreet behaviours, such as a glance, altered demeanour or avoidance of discussions about HIV. Additionally, older adults reported experiencing an unwelcome sense of curiosity from healthcare providers concerning modes of HIV transmission.

It was just a couple of years ago when I was undergoing some examination, and there was this nurse who popped her head in and asked, “How did you get HIV?” (3)

Such experiences undermined their sense of personal integrity and contributed to feelings of inferiority. They also sometimes experienced curiosity from healthcare providers regarding their sexual identities.

From the perspective of older adults living with HIV, more knowledge is needed to diminish the likelihood of discrimination, judgement or prejudice.

I go to the healthcare center, with my other problems and no one has ever reacted to it…. I've never felt like I've been treated in any special way because I have HIV. (9)

When HIV status was treated as just one aspect of their medical profile, they felt acknowledged by the healthcare professionals and not like HIV impacted their healthcare experience.

## 6 Discussion

This study's findings illustrate the complex and sometimes ambiguous healthcare experiences and needs of older adults living with HIV in Sweden. Despite the current year being 2024, the findings revealed persisting experiences of stigma within healthcare, including encounters marked by fear from healthcare professionals. The combined impact of HIV‐related stigma alongside ageism, or other forms of marginalised identities, such as being born abroad, so‐called intersectional stigma can amplify marginalisation and health disparities among people living with HIV (Jackson‐Best and Edwards 2018). Thus, there is a need to improve knowledge and awareness of HIV and intersectional stigmas in healthcare to enhance treatment, reduce negative attitudes and promote quality care for older adults living with HIV. Moreover, the findings suggest that older adults living with HIV benefit from receiving care at HIV‐dedicated clinics, which have more expertise in managing the disease and treating people with HIV. The older adults in this study reported challenges when receiving care from healthcare providers outside these specialised clinics, as those providers tend to have less experience in disease management and in working with individuals living with HIV. From patients' point of view, it is important to be treated with respect, professionality and to be listened at, something that could be assumed not only valid for people living with HIV, but for all people in healthcare encounters (Williams‐Roberts, Abonyi, and Kryzanowski 2018).

These findings highlight the multifaceted vulnerability experienced by patients in healthcare interactions, which is exacerbated by stigmatisation and negative attitudes. Healthcare professionals' attitudes and approaches play a crucial role in this dynamic, because failures in their attitudes have been linked to negative patient experiences, as negative attitudes have been linked to poor patient experiences. Ineffective communication behaviours are a common source of patient dissatisfaction (Råberus et al. 2018). People living with HIV, who often anticipate encountering prejudice, can dissuade themselves from disclosing their HIV status to others (Reynolds et al. 2022). This emphasises the significance of healthcare providers' attitudes and behaviours in shaping patient perceptions and outcomes. Moreover, this underscores the significance of upholding fundamental human rights for people living with HIV. Article 25 of the Universal Declaration of Human Rights (United Nations (UN) 1948) emphasises the right to health and healthcare. Therefore, healthcare should be provided to individuals living with HIV without discrimination, thereby preserving their dignity and rights. Upholding dignity, as described by Delmar (2013), balances patients' expectations and values. This can be achieved by applying a person‐centred approach, which calls for empathy, respect, engagement and relational aspects such as communication in interactions with patients (Håkansson Eklund et al. 2019). However, there are potential pitfalls in patient communication that can result in communication that is unnecessarily harmful (Westendorp et al. 2022). Thus, healthcare professionals' communication skills must be improved or harmful communication approaches de‐implemented to overcome traditional practices and sceptical attitudes towards the realisation of person‐centred care (Moore et al. 2017). It is essential to be aware of the importance of using respectful language and behaviours to be empathic, showing sensitivity in communication and avoiding further increasing HIV‐related stigmatisation (UNAIDS 2015b). Thus, communication and behaviours in line with a person‐centred approach must not be neglected in the care and treatment of older adults living with HIV.

The current findings show that there is still a lack of knowledge about HIV, ART and nonrisks of HIV transmission among healthcare providers. In 2008, the release of the Swiss Statement, a landmark document in the field of HIV research and public health policy, stated that individuals living with HIV who maintain an undetectable viral load through effective ART are highly unlikely to transmit the virus to their sexual partners (Vernazza, Bernasconi, and Flepp 2008). This statement is supported by evidence from clinical trials and observational studies demonstrating the lack of risk of HIV transmission in individuals with sustained viral suppression—Undetectable = Untransmittable (U = U) (Cambiano et al. 2019). However, the lack of knowledge illustrated in this study and others poses risks such as ineffective communication with patients, missed opportunities for prevention and potential stigmatisation and discrimination, which may lead to suboptimal patient outcomes (Bor et al. 2021). A recent scoping review revealed that geriatricians felt uncomfortable and lacked experience in managing older people living with HIV. It showed that there were significant knowledge gaps regarding HIV management, and geriatricians expressed a strong need for more training and specific clinical guidelines (Jones and Barber 2022). Thus, it is important for an understanding about HIV transmission to be widely disseminated, also highlighted by the participants in our study. Integrating care services that address both HIV and age‐related health issues is crucial as having knowledge of healthcare is crucial for preventing discrimination and promoting well‐being, as it enables healthcare professionals to offer informed, sensitive and equitable person‐centred care tailored to the needs of everyone. Also, public awareness campaigns and educational programmes can help change perceptions, reduce discrimination and improve the social environment for older adults living with HIV.

In this study, participants underscored the importance of being involved in their care and treatment and of being able to trust healthcare professionals and the received healthcare. Similarly, a review identified that patients valued a good healthcare professional–patient relationship, emphasising qualities such as compassion and approachability, access to specialised HIV knowledge, continuity of care and involvement in treatment decisions (Cooper et al. 2016). The older adults in our study had the opportunity to demonstrate their competence through support and professional guidance and could actively participate in making health‐related decisions. This is in line with a person‐centred approach emphasising shared decision‐making between patients and healthcare professionals, in which both parties collaborate to make informed choices about treatment options and care plans (Ekman 2022). Moreover, a person‐centred approach means recognising and acknowledging patients as capable coproducers of their care (Entwistle and Watt 2013). Involving older adults living with HIV in decision‐making processes can significantly enhance their sense of responsibility, autonomy and engagement in their care, ultimately improving their overall healthcare experience, healthcare delivery and patient outcomes. However, current practices often face challenges in achieving this alignment, and more knowledge on how to ensure that all patients receive person‐centred care is urgently needed. Stakeholders need to drive initiatives for implementing targeted strategies in healthcare to improve the overall well‐being and quality of life for individuals ageing with HIV, ensuring they receive the care and support they need.

### 6.1 Strengths and Limitations

This study had several methodological considerations. The chosen qualitative approach is beneficial when exploring lived experiences (Sundler et al. 2019), as in the current study. This study provides an in‐depth understanding of older adults' healthcare experiences and valuable insights needed to improve healthcare services. Scientific rigour and validity of qualitative findings can be judged based on how the research process is presented and whether findings and conclusions are sound and well‐founded (Sundler et al. 2019). Additionally, findings in such studies are not aimed at applying to the population at large (Malterud 2001). The findings illustrate important insights needed to understand the needs of older adults living with HIV and their healthcare experiences. It is possible that the findings could be transferred to similar contexts and other older adults in similar ages in countries with corresponding healthcare systems like Sweden, where this study was conducted.

Another limitation is the inclusion of healthy participants in the study. This may also affect the transferability of the findings to all older adults living with HIV, as this study may miss important insights that can only be obtained from those with the most healthcare needs or with dementia. However, further studies are required to test this hypothesis. To create a more inclusive and engaging research environment for older individuals, it is important to involve both frail older adults living with HIV, advocates and health professionals in designing and planning the study, tailoring communication and offering participation opportunities and consider the involvement of caregivers or family members (Goodwin et al. 2023). However, this study included many participants with varying socioeconomic backgrounds and capabilities.

### 6.2 Recommendations for Further Research

Further research is required to explore the experiences and needs of older adults with higher levels of frailty. This could mean involving advocates for this group, such as family members or healthcare professionals, as study participants.

More studies are needed to investigate intersectional stigma on older adults and how healthcare services should address these issues. Strategies for a more person‐centred approach can be beneficial as it empowers patients to engage in their care, increases their autonomy, and enhances their overall healthcare experience. Current findings suggest gaps in the implementation of such strategies in healthcare (Moore et al. 2017). Adopting guidelines with more person‐centred care and education initiatives on how to put person‐centred care and communication into practice requires further development and research. These initiatives then need to be evaluated, both for its impact on quality of care, but also its impact on older adults living with HIV's health outcomes and experiences of care and healthcare needs.

### 6.3 Implications for Policy and Practice

This study highlights the importance of incorporating the expertise and perspectives of older adults living with HIV into the development and planning of HIV care and treatment, both within and outside of HIV‐specific clinics. Additionally, the findings emphasise the need for ongoing efforts by medical and educational institutions to train healthcare providers outside of HIV clinics in managing the disease and treating individuals with HIV. This is essential to actively decrease intersectional stigmatisation, marginalisation and health disparities among people living with HIV.

Further debate, education and research are necessary to improve attitudes towards people living with HIV and to develop the person‐centred communication skills required to prevent stigmatisation by healthcare providers.

## 7 Conclusion

The current findings suggest an urgent need for further efforts to destigmatise HIV and prioritise individualised patient‐centred approaches in healthcare interactions. By addressing these challenges, healthcare delivery can improve the overall well‐being and health outcomes of older adults living with HIV. There is a crucial need for more knowledge on HIV, ART, and transmission, particularly concerning how HIV affects ageing. It is even more critical for healthcare professionals to enhance their communication skills. This improvement should focus not only on interactions with people living with HIV but also on communication among various healthcare facilities. Enhanced communication skills lead to better decision‐making and foster a sense of partnership in care, ensuring that older adults feel that the healthcare system collectively takes responsibility for their treatment and well‐being.

## Author Contributions

**Marie Lund:** data collection, preliminary and final analysis, preparation of and writing the original draft and writing, reviewing and editing the manuscript. **Annelie J. Sundler:** conceptualisation, methodology, data collection, final analysis and writing, reviewing and editing the manuscript. **Ewa Carlsson Lalloo:** project administration, conceptualisation, methodology, data collection, final analysis and writing, reviewing and editing the manuscript.

## Conflicts of Interest

The authors declare no conflicts of interest.

## Supporting information


File S1

## Data Availability

The participants of this study did not give written consent for their data to be shared publicly, so due to the sensitive nature of the research supporting data is not available.
